# Long-Term Safety and Efficacy of Lacosamide Combined with NOACs in Post-Stroke Epilepsy and Atrial Fibrillation: A Prospective Longitudinal Study

**DOI:** 10.3390/jpm14121125

**Published:** 2024-11-27

**Authors:** Marilena Mangiardi, Francesca Romana Pezzella, Alessandro Cruciani, Michele Alessiani, Sabrina Anticoli

**Affiliations:** 1 Department of Stroke Unit, San Camillo-Forlanini Hospital, 00152 Rome, Italy; frpezzella@gmail.com (F.R.P.); sabrina.anticoli@gmail.com (S.A.); 2 Unit of Neurology, Neurophysiology, Neurobiology, Department of Medicine, Campus Bio-Medico University, 00128 Rome, Italy; alessandrocruciani@unicampus.it; 3 Neurology Division S. Maria Goretti Hospital, 04100 Latina, Italy; michelealessiani@gmail.com

**Keywords:** atrial fibrillation, post-stroke epilepsy, post-stroke seizures, ischemic stroke, non-vitamin K antagonist oral anticoagulants (NOACs)

## Abstract

**Background and Aims:** Stroke is the leading cause of seizures and epilepsy in adults; however, current guidelines lack robust recommendations for treating post-stroke seizures (PSSs) and epilepsy (PSE). This study aims to demonstrate the long-term safety and efficacy of lacosamide combined with non-vitamin K antagonist oral anticoagulants (NOACs) in patients with PSE and atrial fibrillation (AF). **Methods:** In this prospective longitudinal single-center study, 53 patients with concomitant PSE and AF, admitted between 2022 and 2023, received NOACs for AF management and lacosamide for seizure control. A control group of 53 patients with cardioembolic stroke, receiving NOACs (but without PSE), was matched by age, sex, and NIHSS scores to ensure comparability. **Results:** Over 24 months, 16 patients in the study group and 15 in the control group experienced new embolic events, with no significant difference between groups (*p* = 0.82). Seizure control improved significantly in the study group, with reduced frequency and severity. No severe adverse events from lacosamide were observed. **Conclusions:** The combination of NOACs and lacosamide is a safe and effective treatment for patients with AF and PSE and does not increase the risk of recurrent ischemic or hemorrhagic events. Further studies with larger sample sizes and longer follow-ups are needed to confirm these findings and optimize treatment protocols.

## 1. Introduction

Ischemic/hemorrhagic stroke is one of the leading causes of morbidity and mortality globally and represents the primary precipitant event for seizures and epilepsy in adults. Between 2 and 14% percent of ischemic stroke survivors develop post-stroke epilepsy (PSE), while the incidence ranges from 10% to 20% following hemorrhagic stroke, as reported by Feyssa et al. [1]. The latency period for PSE onset varies, with 40–80% of cases emerging within the first year after the ischemic/hemorrhagic event. PSE is a significant determinant of stroke prognosis, associated with higher mortality rates, poorer outcomes, and prolonged hospital stays [1]. The occurrence of seizures after a stroke event, commonly referred to as post-stroke seizures (PSSs), negatively impacts recovery and quality of life. When seizures recur over time, this condition may evolve into post-stroke epilepsy (PSE) [2]. Understanding the distinction between these two conditions and their management is crucial for improving patient outcomes.

### 1.1. Post-Stroke Seizures (PSSs)

Post-stroke seizures are relatively common, occurring in a notable subset of stroke survivors [3,4]. These seizures can be classified based on their timing:

1. Early Seizures: Occurring within the first week after a stroke event, these are typically provoked by the acute effects of the stroke, such as brain injury, hemorrhage, or metabolic disturbances.
2. Late Seizures: These occur after the first week post-stroke and are considered more likely to indicate the development of epilepsy.

The pathophysiology of PSS involves acute neuronal injury, excitotoxicity, and metabolic changes, which lead to hyperexcitability in the affected brain regions. Hemorrhagic strokes, large cortical infarcts, and specific stroke subtypes are more likely to result in early seizures [5]. One of the primary mechanisms is acute neuronal injury, which occurs when the blood flow directed to a specific brain area is interrupted, thus leading to cell death and tissue damage. This injury can cause the release of excitatory neurotransmitters, such as glutamate, in excessive amounts, a process known as excitotoxicity [6]. This stress is characterized by an imbalance in the levels of oxygen and glucose, which are vital for normal cell function [7]. The lack of oxygen (hypoxia) and glucose (hypoglycemia) supply can impair mitochondrial function, leading to energy failure and the accumulation of metabolic by-products that contribute to neuronal hyperexcitability [8]. Possibly, also, the genetic susceptibility such as the mitochondrial aldehyde dehydrogenase 2 (ALDH2) polymorphism is associated with PSS development [9].

### 1.2. Post-Stroke Epilepsy (PSE)

When a patient experiences recurrent seizures beyond the acute phase of stroke recovery, the condition is termed PSE. PSE is a form of secondary epilepsy, where the stroke acts as the initial insult that triggers chronic epileptogenic processes [10].

### 1.3. Factors Contributing to PSE Development

- Stroke Severity: Larger and more severe strokes have a higher risk of resulting in PSE. Lacunar strokes, which involve small, deep brain structures, generally have a lower risk of leading to seizures, unless they are extensive or involve critical pathways [11].
- Stroke Type: Hemorrhagic strokes carry a greater risk compared to ischemic strokes. The presence of blood in the brain parenchyma can act as an irritant, disrupting normal neuronal activity and creating an environment favorable to seizures. Additionally, hemorrhagic strokes can lead to the breakdown of the blood–brain barrier, allowing substances that are normally excluded from the brain to enter and cause inflammation and further neuronal damage [11].
- Location: Cortical involvement significantly increases the risk of developing PSE [12].
- Early Seizures: The presence of early post-stroke seizures is a strong predictor of subsequent epilepsy [13] (Table 1).

### 1.4. Clinical Implications and Management

#### Role of EEG in the Diagnosis and Prediction of Post-Stroke Seizures

The diagnosis of PSSs and PSE requires a detailed medical history, neurological examination, and appropriate imaging studies to identify the lesion and its aftermath. Electroencephalography (EEG) is essential for detecting abnormal electrical activity, indicative of seizures or epilepsy, assessing the validity of epileptic seizures during interictal periods, estimating initial seizure risk after stroke, evaluating further seizure recurrence risk, and predicting outcomes [14,15].

The American Clinical Neurophysiology Society’s Standardized Critical Care EEG Terminology suggests that specific interictal EEG patterns can indicate a heightened risk of epileptic seizures [16]. Recent EEG studies have demonstrated that interictal epileptiform discharges (IEDs), periodic discharges (PDs), and rhythmic delta activity (RDA) are commonly observed in patients with PSE. Specifically, as reported by Brophy et al. [17], IEDs were found in 29.8% of patients, PDs in 19.2%, and RDA in 17.6%. These findings highlight the importance of EEG monitoring in identifying patients at increased risk of seizures and guiding appropriate clinical interventions. A prospective study in acute stroke patients found that interictal epileptiform discharges (IEDs) and asymmetry in background activity were the most significant predictors of subsequent late seizures. In contrast, periodic discharges (PDs) and rhythmic delta activity (RDA) did not significantly predict future seizures [18,19].

The distinction between early and late seizures is fundamental in clinical practice. A seizure occurring within a week of a stroke is considered early and acutely symptomatic. While these seizures carry a certain risk of subsequent epilepsy, they do not warrant a diagnosis of PSE. In contrast, a seizure occurring more than a week after a stroke is considered late and unprovoked, with a recurrence risk greater than 60%, thus meeting the diagnostic criteria for epilepsy [20].

In some situations, the distinction between early and late seizures may not be easy, especially if the clinical picture is unstable or the exact timing of the cerebral insult is not well defined. The diagnosis of epilepsy requires a recurrence risk exceeding 60%, but it can be challenging to estimate this risk precisely. In cases of uncertainty, it is preferable not to diagnose a late seizure or PSE, adopting a watchful waiting approach instead. The decision of initiating treatment with antiepileptic drugs will depend on the individual clinical characteristics of each patient. EEG is an essential diagnostic tool in the early stages following an ischemic or hemorrhagic stroke. EEG can detect purely electrographic seizures and specific patterns, such as lateralized periodic discharges (LPDs), which are independently associated with early seizures [21]. Single-photon emission computed tomography (SPECT) imaging can reveal focal hypermetabolism with increased cerebral blood flow associated with LPDs, suggesting that, in some patients, this EEG pattern may correspond to an ictal phenomenon. The lack of systematic electrophysiological assessment with video-EEG can lead to an underestimation of seizures, particularly in the case of focal unaware or non-convulsive seizures. Neurologists and healthcare providers in stroke units should promptly request EEG recordings for patients with sudden unexplained behavioral changes or alterations in consciousness. Continuous EEG monitoring of at least 24 h should be initiated as soon as possible in patients with acute supratentorial brain injuries presenting with altered mental status or clinical paroxysmal events suspected to be seizures [22]. In comatose patients, those with periodic discharges, or those pharmacologically sedated, prolonged EEG monitoring (≥48 h) can detect non-convulsive seizures. EEG may also have implications in predicting functional outcomes, mortality, and post-stroke cognitive decline. Although few studies have evaluated EEG as a predictive tool for post-stroke seizures and epilepsy, EEG abnormalities can predict the development of epilepsy within the first year after a stroke, independently of clinical and imaging-based infarct severity [23,24].

### 1.5. Post-Stroke Epilepsy and Antiepileptic Medication Management

In managing post-stroke epilepsy, primary antiepileptic medication (ASM) prophylaxis is not recommended due to insufficient evidence of any reduction in acute symptomatic or unprovoked seizures or improvement in functional outcomes and mortality rates in different studies [25]. Instead, short-term ASM treatment (1–4 weeks) is used for acute symptomatic or early seizures. The risk of seizure recurrence after an acute symptomatic seizure is low (10–20%), making long-term ASM use unnecessary unless unprovoked seizures occur, where the recurrence risk can exceed 60%.

Long-term ASM use is typically reserved for unprovoked seizures due to a higher 10-year recurrence risk (70%) [26,27]. Table 2 summarizes the main indications for the short- and long-term use of ASMs in primary and secondary prophylaxis in patients with PSE.

The choice of ASM should be individualized, considering patient factors, like stroke type, comorbidities, and potential drug interactions [28,29]. Commonly used ASMs include lamotrigine, carbamazepine, lacosamide, levetiracetam (LEV), phenytoin, and valproate.

Lamotrigine, levetiracetam, and lacosamide are preferred due to their tolerability and efficacy, with levetiracetam being a common choice despite debates about its interaction with direct oral anticoagulants (DOACs) [30,31]. However, studies show that levetiracetam has a minimal impact on DOAC levels compared to enzyme-inducing ASMs (e.g., carbamazepine, phenytoin).

Lamotrigine has demonstrated higher efficacy as a first-line treatment for focal epilepsy compared to levetiracetam and zonisamide [32,33]. Levetiracetam, commonly used in post-stroke epilepsy, may induce P-glycoprotein (P-gp), potentially interacting with DOACs. However, the role of levetiracetam as a P-glycoprotein inducer in humans remains debated. Study results are conflicting; some data support the combination of non-vitamin K oral anticoagulants (NOACs) and levetiracetam, suggesting that levetiracetam does not alter the plasma levels of NOACs [11,34].

A recent study published examined the effects of levetiracetam (LEV) compared to enzyme-inducing ASMs on the peak plasma concentrations (Cmax) of apixaban and rivaroxaban [35]. Patients on LEV did not show significant reductions in these anticoagulants’ Cmax, in contrast to those treated with enzyme-inducing ASMs like carbamazepine or phenytoin [35].

Lacosamide is a well-tolerated and effective treatment for epilepsy of cerebrovascular origin, including post-stroke epilepsy. It reduces seizure frequency similarly to carbamazepine but with fewer side effects, making it a preferable option for many patients. Lacosamide stabilizes hyperexcitable neuronal membranes by selectively enhancing the slow inactivation of voltage-gated sodium channels, reducing seizure risk without broadly suppressing normal neuronal activity. This mechanism, along with its favorable safety profile, supports its use in managing epilepsy, particularly in vulnerable populations like those with ischemic stroke [36,37].

### 1.6. Optimizing Anticoagulant and Antiseizure Medication Combinations: Choosing the Best Approach

In patients with atrial fibrillation (AF) and history of stroke, NOACs are often prescribed to prevent further thromboembolic events. The combination of NOACs and ASMs, such as lacosamide, has been proven safe and effective, without increasing the risk of recurrent ischemic or hemorrhagic events [37].

DOACs, also known as NOACs, comprise a group of five drugs that directly target the coagulation cascade without relying on anti-thrombin as a mediator [38]. These include factor Xa inhibitors (apixaban, edoxaban, and rivaroxaban) and direct thrombin inhibitors (argatroban and dabigatran). NOACs are primarily indicated for anticoagulation in the prevention and treatment of ischemic stroke in patients with non-valvular AF [39,40]. They are also widely used for the prevention and management of pulmonary embolism and deep venous thrombosis [41].

Strokes and other cerebrovascular diseases are the leading causes (30–40%) of symptomatic epilepsy in the elderly population [42]. Many elderly patients with stroke require long-term ASM [43]. Consequently, it is not uncommon for these patients to receive concurrent treatment with both ASMs and NOACs, a combination that raises the potential for significant pharmacological interactions, with serious health-related implications [44]. Specifically, ASMs which reduce NOACs absorption or increase their metabolism can diminish the antithrombotic effect of NOACs. Differently, reduced metabolism of NOACs can substantially increase the bleeding risk. Addressing drug–drug interactions (DDIs) is critical in epilepsy management, as achieving optimal seizure control often needs various treatment adjustments or ASM polytherapy [43]. Pharmacokinetic interactions between ASMs and other medications primarily involve the cytochrome P450 (CYP) enzyme system and, to a lesser degree, the uridine diphosphate glucuronosyltransferase (UGT) system. First-generation ASMs like carbamazepine, phenytoin, and phenobarbital are strong inducers of several CYP enzymes, as well as UGTs and epoxide hydrolase. In contrast, valproate inhibits CYP2C9 and other enzymes, while newer ASMs such as perampanel and oxcarbazepine have weaker enzyme-inducing effects. Some ASMs, including LEV and zonisamide, interact with P-gp, potentially affecting drug transport and metabolism. Currently, there are no published data on the induction or inhibition of CYP or UGT isoenzymes by ethosuximide, lacosamide, gabapentin, pregabalin, or vigabatrin [45,46]. The overall impact of these interactions varies, with newer ASMs having fewer complications when compared to older-generation drugs. Table 3 shows the interactions between various ASMs and P-gp as well as CYP3A4/3A5 and CYP2J2 enzyme systems.

There is no consensus on the use of prophylactic ASMs for preventing PSS or PSE, particularly in the absence of early seizures. The decision to initiate ASMs prophylactically should be based on the clinical context and individual risk factors [44].

The current guidelines, indeed, do not clearly define the best approach for combining anticonvulsant drugs with oral anticoagulants. As a result, clinicians must rely on their experience, understanding of pharmacokinetics and pharmacodynamics, and the individual patient’s characteristics—such as age, risk factors, and comorbidities—to determine the most appropriate combination therapy. Given that enzyme-inducing or -inhibiting anticonvulsants are often less predictable in this patient group and can affect the plasma levels of oral anticoagulants, we assessed the efficacy and safety of lacosamide when used alongside NOACs, leveraging its well-established and potentially safer pharmacokinetic profile.

### 1.7. Aim of the Study

This prospective longitudinal single-center study was conducted to evaluate the safety and efficacy of lacosamide in combination with NOACs in patients admitted to the Stroke Unit Department with concomitant PSE and AF, between September 2022 and May 2023. After being discharged from the ward, patients were followed up in the outpatient clinic of the same department for 24 months, each patient undergoing a total of four consecutive neurological visits (every 3 months apart). Data collection was conducted according to the guidelines of the Declaration of Helsinki and approved by the Institutional Ethics Committee. Inclusion and exclusion criteria are summarized in Table 4.

### 1.8. Study Design and Population

Study Group: The study group consisted of 53 patients diagnosed with concomitant PSE and AF. Each patient in this group was administered NOACs to manage AF and lacosamide to control seizures. The choice of NOACs was based on individual patient profiles, considering factors, such as renal function, bleeding risk, and patient preference. Lacosamide, a newer ASM known for its efficacy and safety profile, was selected to address the seizure activity. Among the 53 patients included in the study group, all were treated with a combination of NOACs and lacosamide for managing AF and PSE, respectively. Below is a detailed breakdown of the specific treatments and clinical profiles:

### 1.9. NOAC Subtypes Administered

- Apixaban: 21 patients.
- Rivaroxaban: 12 patients.
- Dabigatran: 10 patients.
- Edoxaban: 10 patients.

### 1.10. Antiseizure Medication (ASM) Treatment

- Lacosamide: All 53 patients in the study group received lacosamide for seizure control, with an average dose of 100 mg BID. Lacosamide was introduced as the first-line antiepileptic drug at the time of PSE diagnosis. For better seizure control, it was necessary to add an additional antiepileptic drug for some patients, as detailed below.
- Combination Therapy:
  ○ Perampanel: 8 patients received perampanel at a dose of 4 mg/day in combination with lacosamide.
  ○ Brivaracetam: 10 patients were treated with brivaracetam at a dose of 100 mg BID in conjunction with lacosamide.

### 1.11. Seizure Subtypes

The majority of patients experienced focal seizures with or without secondary generalization, a common presentation of PSE. These seizures were characterized by localized onset, often with motor or sensory symptoms, and occasionally progressed to generalized seizures. Of the 53 patients in the study group, 21 experienced one or more seizures within the first week following stroke onset, leading to a diagnosis of post-stroke seizure (PSSs). Specifically, among the 21 patients initially diagnosed with PSS, 20% had generalized seizures, 45% had focal seizures with or without secondary generalization, 15% experienced myoclonic seizures, and 5% had status epilepticus. The remaining cases involved seizures of unknown morphology. All of these patients developed PSE that was diagnosed based on clinical criteria (at least one unprovoked seizure occurring one week or more, after the diagnosis of ischemic stroke) and supported by electroencephalographic monitoring. Specifically, in the study group, 32 patients experienced one or more unprovoked seizures within a timeframe of 8 to 12 days after the stroke diagnosis; 10 patients had one or more unprovoked seizures between 20 and 30 days; and the remaining 11 patients within 60 days. The seizure subtypes were focal seizures with/without secondary generalization.

### 1.12. Stroke Subtypes

**Figure 1:**
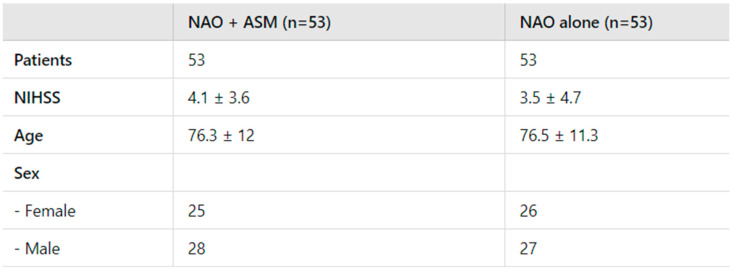
Main characteristics of the two groups of patients matched for age, sex, and NIHSS score. *p* = 0.462.

All patients enrolled in this study had a prior history of ischemic stroke, with the totality of cases being cardioembolic strokes, which are commonly associated with AF. Stroke classification was based on clinical evaluation, brain imaging (MRI or CT), and relevant clinical history. At stroke onset, the NIHSS scores of the 53 patients in the study group were high or moderate, ranging between 10 and 18, with an average score of approximately 12.5. Notably, 80% (n = 42) of the patients who developed post-stroke epilepsy (PSE) in the study group had a middle cerebral artery (MCA) occlusion and were treated with thrombolysis and/or thrombectomy, according to the most recent guidelines. The remaining 20% (n = 11) of patients, who did not have evidence of MCA occlusion, were diagnosed with moderate cardioembolic stroke. The NIHSS scores reported for the study group in Figure 1 reflect the values recorded at discharge from the stroke unit and were compared with the NIHSS scores of the control group.

Control Group: A control group comprising 53 patients with cardioembolic stroke was established. These patients were receiving NOACs as part of their standard treatment protocol. The control group was meticulously matched to the study group based on several parameters, including age, sex, and mean National Institutes of Health Stroke Scale (NIHSS) scores, to ensure comparability and minimize confounding variables (Figure 1). The NOAC subtypes administered in the control group were the following:

- Apixaban: 18 patients.
- Rivaroxaban: 15 patients.
- Dabigatran: 10 patients.
- Edoxaban: 10 patients.

The characteristics of the study population are summarized in Table 5.

### 1.13. Objectives

Primary outcome: The primary outcome of this study was to establish the following:

1. Efficacy of Seizure Control: The effectiveness of lacosamide in managing and controlling seizures in patients with PSE.
2. Safety Profile: The safety of the combination therapy particularly focuses on the recurrence rate of minor or major embolic events over a 24-month follow-up period. This included monitoring for ischemic strokes, hemorrhagic strokes, and other embolic complications [Venous Cerebral Sinus Thrombosis, Transient Ischemic Attack (TIA), Ischemic Stroke or Minor Stroke, Peripheral Venous Thrombosis].

## 2. Methodology

### 2.1. Patient Monitoring and 24-Month Follow-Up

Patients were monitored regularly through clinical visits, telemedicine consultations, and routine diagnostic tests, including EEG and MRI, to track seizure activity and detect any new embolic events. Patients’ adherence to the medication regimen was reinforced through regular counseling sessions, and potential side effects were meticulously recorded and managed.

### 2.2. Data Collection and Analysis

Detailed patient records were maintained, documenting demographic data, stroke characteristics, treatment regimens, and outcomes. Statistical analysis was performed to compare the incidence of embolic events and seizure control between the study and control groups. Kaplan–Meier survival analysis and Cox proportional hazards models were used to evaluate time-to-event data and identify predictors of recurrent embolic events. Mean, standard deviation, and proportions were calculated to summarize the baseline characteristics of the study population. Chi-square tests and Fisher’s exact tests were employed to compare categorical variables (e.g., sex, incidence of embolic events) between the groups. Independent *t*-tests were used to compare continuous variables (e.g., age, NIHSS scores). Multivariable Cox regression models were constructed to identify predictors of recurrent embolic events while adjusting for potential confounders. Hazard ratios (HRs) with 95% confidence intervals (CIs) were calculated to quantify the association between variables (e.g., treatment group, age, stroke severity) and the risk of recurrent embolic events. Repeated measures ANOVA was employed to compare seizure control metrics between the study and control groups across multiple time points.

## 3. Results

**Figure 2:**
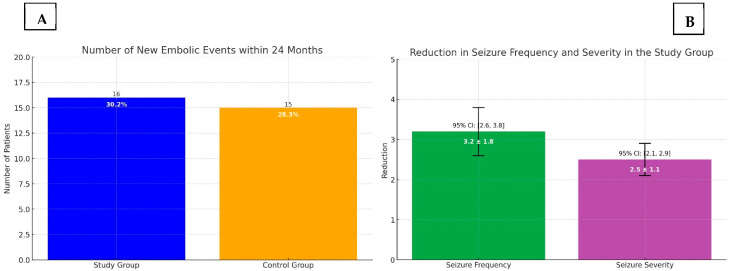
Panel (**A**) New embolic events within 24 months: Histogram illustrating the number of new embolic events within 24 months for both the study group and the control group. The percentages of patients experiencing embolic events are also displayed on the bars for clearer comparison. In blue the study group population; in orange the control group population. Panel (**B**) Reduction in seizure frequency and severity within 24 months: Reduction in seizure frequency and severity in the study group. The bars display the mean reduction, and the error bars represent the 95% confidence intervals. In green the seizure frequency rate, in purple the seizure severity in the study group.

In the study group, 16 out of 53 patients (30.2%) experienced a new embolic event within 24 months, compared to 15 out of 53 patients (28.3%) in the control group. The difference between the two groups was not statistically significant (*p* = 0.82). The Kaplan–Meier survival analysis showed similar survival curves for both groups (log-rank test, *p* = 0.78), indicating that the combination of NOACs and lacosamide did not increase the risk of recurrent ischemic or hemorrhagic events in patients with AF and PSE. Seizure control was effectively achieved in the majority of patients in the study group, with a significant reduction in seizure frequency and severity reported during the follow-up period. The mean reduction in seizure frequency was 3.2 ± 1.8 episodes per month (*p* < 0.01, 95% CI: 2.6 to 3.8), and the severity of seizures, measured using a standardized seizure severity scale, decreased by 2.5 ± 1.1 points (*p* < 0.01, 95% CI: 2.1 to 2.9). No severe adverse effects attributable to lacosamide were observed, reinforcing its safety profile when used alongside NOACs. Only one patient (1.9%) developed a second-degree atrioventricular block after taking lacosamide in the study group. Consequently, lacosamide was discontinued, and the patient was withdrawn from this study. Figure 2 summarizes the number of new embolic events along the 24-month follow-up (Figure 2A) and the reduction in seizure frequency and severity (Figure 2B) in the study group.

## 4. Discussion

This study demonstrated that lacosamide, in conjunction with NOACs, is a safe and effective treatment strategy for patients with concomitant PSE and AF, followed-up for 24 months. The absence of an increased risk of embolic events highlights the potential of this therapeutic combination to provide comprehensive management of both seizure activity and stroke prevention in this high-risk population. Also, combining lacosamide with NOACs can offer dual benefits: reducing the frequency and severity of seizures while also preventing recurrent ischemic events. This dual approach is particularly beneficial for patients with AF, who are at increased risk of stroke, and for those with PSE, where seizure control is paramount to improve quality of life. The study findings underscore the importance of safety when managing patients with comorbid conditions. The fact that no significant increase in embolic events was observed is crucial, as it reassures healthcare providers about the safety of adding lacosamide to the treatment regimen of patients already taking NOACs. This is significant given the delicate balance required to manage anticoagulation in patients at risk of both seizures and thromboembolic events. While the results are promising, several limitations must be acknowledged:

Sample Size: The relatively small sample size of 53 patients in each group limits the generalizability of the findings. Larger trials are needed to validate these results and ensure they are applicable to a broader population.

Follow-Up Duration: The follow-up period of 24 months may not be sufficient to capture all long-term outcomes and potential delayed adverse effects. However, stroke recurrences occur most often within the first two years from first stroke onset [47]. Extended follow-up is necessary to assess the sustained efficacy and safety of the treatment combination.

Single-Center Design: As this study was conducted at a single center, there may be site-specific factors that influenced the results. Multicenter studies would help mitigate this limitation and provide more robust data.

Real-World Applicability: The controlled clinical environment of this study may not fully reflect real-world clinical practice. Patient adherence, comorbid conditions, and other variables in everyday clinical settings could impact the outcomes.

Future studies with larger sample sizes and longer follow-up periods are warranted to confirm these data and further refine treatment protocols.

Also, future studies should aim to:

- Validate findings: larger, multicenter trials are needed to validate these initial findings and ensure they are generalizable across different populations.
- Acquire data on longer time intervals: longer follow-up periods will help assess the long-term safety and efficacy of the lacosamide and NOAC combination, including any delayed adverse effects or changes in seizure control and stroke prevention over time.
- Increase the number of comparative studies: research comparing different NOACs and antiseizure medicaments (ASMs) will be valuable in identifying the most effective and safest combinations. This approach could lead to more personalized treatment strategies, optimizing outcomes based on individual patient profiles.

Exploring the impact of different NOACs and ASMs on patient outcomes could indeed provide more personalized treatment strategies for individuals with PSE and AF. Personalized medicine, which tailors treatment to the individual characteristics of each patient, has the potential to significantly improve clinical outcomes. Future studies should focus on the following:

- Genetic and biomarker analysis: Identifying genetic markers or other biomarkers able to predict the response to specific ASMs and NOACs can help tailor treatments to individual patients. Recent research has investigated the role of the glymphatic system and perivascular spaces as potential biomarkers for PSE [48,49]. Disruptions in these systems may contribute significantly to the development of epilepsy following a stroke. The findings suggest that abnormalities in the glymphatic system and perivascular spaces could be crucial indicators for the onset and progression of PSE. This discovery offers promising new avenues for early diagnosis and targeted therapy. The research underscores the potential of these biomarkers to enhance the understanding and management of epilepsy in stroke patients, potentially leading to more personalized and effective treatment strategies. Protein expression profiles in patients with PSE significantly differ from those of non-epileptic stroke patients, suggesting the involvement of multiple proteins in the development of post-stroke epileptogenesis. Notably, TNFSF-14 has been identified as a key predictive biomarker for PSE, with its downregulated levels showing strong potential in forecasting the risk of PSE [50,51].
- Drug–drug interactions: Knowledge about the pharmacokinetic and pharmacodynamic DDIs between antiseizure drugs and direct oral anticoagulants is limited, primarily based on in vitro and in vivo studies. Currently, there are no available data on DDIs with NOACs for approximately 67% of ASMs. As the number of patients requiring combined therapy with both is constantly growing, there is a critical need for comprehensive research in this area to inform clinical practice and ensure patient safety. Understanding the pharmacokinetics and pharmacodynamics of different ASMs and NOACs in combination is essential to avoid adverse interactions and optimize therapeutic efficacy [52].
- Patient subgroups: Analyzing outcomes in various subgroups (e.g., age, sex, comorbidities) will help determine which patients are most likely to benefit from specific treatment combinations.
- While the current study presents a positive outlook, it is essential to approach these findings with a degree of caution. The study limitations include the limited sample size and duration. Additionally, real-world applicability may vary, and, thus, ongoing vigilance in monitoring and reporting adverse events in clinical practice is crucial. Moreover, as healthcare systems move towards more integrative and holistic approaches, it is vital to consider the broader context of patient care. This includes the role of lifestyle modifications, patient education, and support systems in managing chronic conditions like PSE and AF. Future research should also explore these aspects to provide a more comprehensive understanding of how best to support patients in achieving optimal health outcomes.

## 5. Conclusions

PSE represents significant challenges in rehabilitation for stroke survivors. Early recognition and appropriate management are critical to improving outcomes and enhancing quality of life for affected individuals. While the current guidelines provide a framework for treatment, ongoing research is essential to refine therapeutic strategies and develop more effective interventions for PSE. This study provides valuable insights into the management of PSE in patients with AF, demonstrating that the combination of lacosamide and NOACs is both safe and effective compared with a control group affected by AF. These preliminary findings pave the way for further research and potential updates to clinical guidelines, aiming to improve the quality of life and clinical outcomes for stroke survivors with PSE.

## Figures and Tables

**Table 1:** Comprehensive overview of various risk factors that can contribute to the development of post-stroke epilepsy.

Risk Factor	Description
Stroke Severity	Larger and more severe strokes increase the likelihood of PSE.
Stroke Type	Hemorrhagic strokes have a higher risk of leading to PSE compared to ischemic strokes.
Cortical Involvement	Strokes affecting the cerebral cortex are more likely to result in PSE.
Early Post-Stroke Seizures	The occurrence of seizures within the first week post-stroke is a strong predictor of later epilepsy.
Age	Younger patients are at a higher risk of developing PSE compared to older patients.
Stroke Location	Strokes in the temporal and frontal lobes are more associated with PSE.
Previous History of Seizures	Patients with a history of seizures before the stroke are more likely to develop PSE.
Stroke Recurrence	Multiple strokes increase the risk of developing PSE.
Genetic Factors	Family history of epilepsy may increase susceptibility to PSE.
Comorbid Conditions	Conditions like hypertension, diabetes, and heart disease can elevate the risk of PSE.
Stroke-Induced Neuroinflammation	Inflammatory responses post-stroke can contribute to the development of epilepsy.
Subarachnoid Hemorrhage	This specific type of hemorrhagic stroke has a high association with PSE.
Intraventricular Hemorrhage	Presence of blood in the brain’s ventricular system can be a risk factor for PSE.
Infectious Complications	Post-stroke infections, especially central nervous system infections, can increase PSE risk.
Blood-Brain Barrier Disruption	Stroke-induced damage to the blood-brain barrier can lead to epileptogenic changes.

PSE, post-stroke epilepsy.

**Table 2:** Indications for the short- and long-term use of ASMs in primary and secondary prophylaxis in patients with PSE based on the current ESO recommendations [28].

Aspect	Detail
Primary ASM Prophylaxis	Not recommended due to insufficient evidence of benefit.
Short-term ASM Treatment	Used for 1–4 weeks for acute symptomatic or early seizures with low recurrence risk.
Secondary ASM Prophylaxis	Not typically required for post-stroke seizures.
Pathophysiological Considerations	Short-term ASM may be used in conditions like reduced brain perfusion, brain edema, or vasospasm.
Long-term ASM Use	Recommended for unprovoked post-stroke seizures due to high recurrence risk.
ASM Discontinuation	Low 10-year risk of unprovoked seizures after one acute symptomatic seizure.
ASM Selection	Should be personalized based on stroke type, patient age, comorbidities, and other factors.
Medication Options	Lamotrigine, carbamazepine, lacosamide, levetiracetam, phenytoin, and valproate, with lamotrigine and levetiracetam being more tolerable.

ASM: antiseizure medications.

**Table 3:** Interactions of antiseizure medications (ASMs) with P-glycoprotein (P-gp) and CYP3A4/CYP3A5/CYP2J2 enzyme systems.

ASMs	P-gp	CYP3A4	CYP3A5/CYP2J2
Eslicarbazepine acetate	Substrate (in vitro)	Weak inductor (in vitro e vivo)	NR
Felbamate	Substrate (in vivo)	Weak inductor/No effects (in vitro)	NR
Gabapentin	Not substrate	NR	NR
Lamotrigine	No effects/substrate	No effects	No effects
Levetiracetam	Inductor/substrate (in vivo)	Weak inductor (in vitro)	No effects
Oxcarbazepine	NR	Inductor (in vivo e vitro)	Inductor 3A5 (in vivo e vitro)
Perampanel	No effects	Weak inductor (in vitro)	Weak inductor 3A5 (in vitro)
Pregabalin	No effects	No effects	No effects
Rufinamide	NR	Mild induction (in vitro)	No effects
Stiripentol	NR	Inhibitor (in vitro)	No effects
Tiagabine	NR	Substrate	No effects
Topiramate	No effects/substrate	Mild inductor (in vitro)	No effects
Lacosamide	No effects	No effects (in vitro)	No effects
Vigabatrin	NR	No effects	No effects
Zonisamide	Weak inhibitor	No effects/substrate	No effects
Phenobarbital	Inductor/substrate	Inductor	No effects
Phenytoin	Inductor/substrate (in vivo)	Inductor/substrate (in vivo)	NR
Ethosuximide	NR	Substrate	NR
Carbamazepine	Inductor (in vivo)	Substrate/inductor (in vitro and vivo)	NR
Valproate	Inductor/inhibitor (in vitro)	Inductor/weak inhibitor (in vitro)	NR

ASMs: Antiseizure medications; P-gp: P-glycoprotein; CYP: Cytochrome P450; NR: Not reported.

**Table 4:** Inclusion and exclusion criteria.

Criteria	Details
Inclusion Criteria	
Age	Patients aged 18 years and older.
Diagnosis of AF	Confirmed AF diagnosis (EKG based) requiring anticoagulation with NOACs.
PSE and Seizure Type	Epilepsy diagnosed post-stroke (PSE) with focal or generalized seizures.
Use of NOACs	NOACs prescribed for AF, subtype chosen based on renal function, bleeding risk, and patient preference.
Stroke Subtype	History of ischemic or cardioembolic stroke, confirmed by clinical and imaging data.
Willingness to Comply	Patients willing to participate, attend follow-ups, and adhere to treatment protocols, including NOAC and lacosamide use.
Informed Consent	Written informed consent obtained from patients or legal guardians.
Exclusion Criteria	
Severe Renal or Hepatic Impairment	Severe renal impairment (creatinine clearance < 30 mL/min) or significant hepatic dysfunction (Child-Pugh C or more).
Hypersensitivity	Known hypersensitivity or allergic reactions to lacosamide or any NOACs.
Other Causes of Epilepsy	Epilepsy due to causes other than stroke (e.g., genetic, traumatic, infectious).
Other Anticoagulation Therapy	Patients requiring anticoagulation with agents other than NOACs (e.g., warfarin, heparin).
Pregnancy or Lactation	Pregnant or breastfeeding women due to potential teratogenic effects.
Uncontrolled Cardiovascular Conditions	Unstable cardiovascular conditions (e.g., decompensated heart failure, severe uncontrolled hypertension).
Significant Drug Interactions	Medications with known interactions with NOACs or lacosamide.
Severe Neurocognitive Impairment	Severe cognitive impairment or dementia, limiting ability to follow protocols or give consent.
Participation in Other Trials	Enrolled in other clinical trials that could interfere with study outcomes or compromise safety.

**Table 5:** Main features of patients enrolled in the study group.

Feature	Details
Total Patients	53
NOAC Subtypes	53 patients total
Apixaban	21 patients
Rivaroxaban	12 patients
Dabigatran	10 patients
Edoxaban	10 patients
Lacosamide Dose (mg/day)	100 mg BID (all patients)
Perampanel (mg/day)	4 mg/day (8 patients)
Brivaracetam (mg/day)	100 mg BID (10 patients)
Seizure Subtype	Focal seizures with/without secondary generalization
Stroke Subtype	Ischemic stroke, mainly cardioembolic

## Data Availability

No new data were created or analyzed in this study. Data sharing is not applicable to this article.
